# Efficient multitasking: parallel versus serial processing of multiple tasks

**DOI:** 10.3389/fpsyg.2015.01366

**Published:** 2015-09-08

**Authors:** Rico Fischer, Franziska Plessow

**Affiliations:** ^1^Department of Psychology, Technische Universität Dresden, Dresden, Germany; ^2^Berenson-Allen Center for Noninvasive Brain Stimulation, Department of Neurology, Beth Israel Deaconess Medical Center, Harvard Medical School, Boston, MA, USA

**Keywords:** multitasking, dual-tasking, crosstalk, psychological refractory period (PRP), cognitive control, functional bottleneck, bottleneck, parallel versus serial processing

## Abstract

In the context of performance optimizations in multitasking, a central debate has unfolded in multitasking research around whether cognitive processes related to different tasks proceed only sequentially (one at a time), or can operate in parallel (simultaneously). This review features a discussion of theoretical considerations and empirical evidence regarding parallel versus serial task processing in multitasking. In addition, we highlight how methodological differences and theoretical conceptions determine the extent to which parallel processing in multitasking can be detected, to guide their employment in future research. Parallel and serial processing of multiple tasks are not mutually exclusive. Therefore, questions focusing exclusively on either task-processing mode are too simplified. We review empirical evidence and demonstrate that shifting between more parallel and more serial task processing critically depends on the conditions under which multiple tasks are performed. We conclude that efficient multitasking is reflected by the ability of individuals to adjust multitasking performance to environmental demands by flexibly shifting between different processing strategies of multiple task-component scheduling.

## Introduction

A central aim in cognitive psychology and cognitive neuroscience research on multitasking is to understand and optimize the underlying processes in order to increase efficiency when dealing with multiple tasks at the same time. Growing demands on information processing due to increasing multimedia interactions call for higher efficiency. Does the human mind have the structural and functional means for efficient multitasking? The architectural properties of the nervous system allow for widely distributed simultaneous neural processing in billions of neurons. However in multitasking, severe performance costs occur when combining even the simplest cognitive tasks for simultaneous execution. Based on the current literature, we answer two key questions of multitasking: *(1) What constitutes efficient and adaptive multitasking? (2) Which processes determine multitasking efficiency?*

Most researchers explain typical performance decrements in multitasking with a structural capacity limitation, a so-called processing bottleneck (e.g., Pashler, 1998), at which certain cognitive processes proceed *serially* (i.e., one at a time only). Such a conceptualization of multitasking excludes the possibility of *parallel* (i.e., simultaneous) cognitive processing of multiple task components^1^. Others argue that parallel processing is generally possible (e.g., by means of capacity sharing, Tombu and Jolicoeur, 2003), but that serial processing reflects the more efficient and thus primarily pursued multitasking strategy (e.g., Logan and Gordon, 2001; Miller et al., 2009). In this review we will outline this debate, specifically focusing on the characteristics of serial versus parallel processing options and how they are theoretically conceptualized as well as empirically assessed in multitasking. For this, we restrict this review on multitasking situations, in which two speeded choice-reaction tasks have to be performed at the same time, i.e., dual tasks (for reviews of sequential multitasking, i.e., task switching, see Monsell, 2003; Kiesel et al., 2010; Koch et al., 2010). We will further elaborate on the question of why people pursue the common aspiration of doing more than one task at a time (engaging in parallel processing) with the intention of increasing their performance efficiency, while serial processing is in fact more efficient. To provide a more complete picture on the debate of parallel versus serial processing, we will also discuss under which circumstances adopting a more parallel processing strategy represents the favorable multitasking solution.

## Capabilities and Limitations of the Cognitive System for Multitasking

### Serial Processing Due to Limited Resources

**FIGURE 1:**
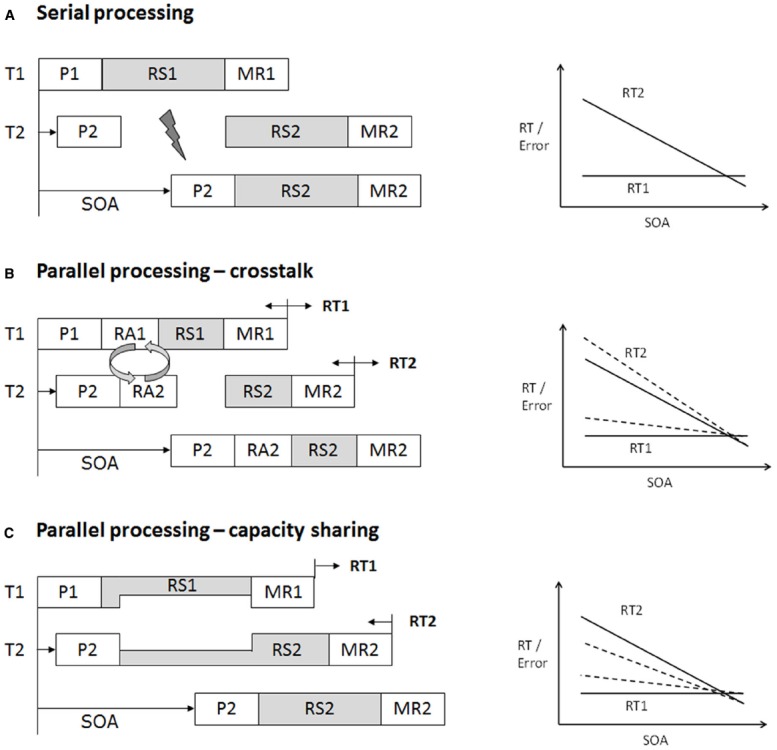
**Schematic illustration of serial task processing (A) and different forms of parallel processing (B,C) of two tasks in the framework of an assumed capacity-limited central processing stage.** Dashed lines illustrate the changes in result patterns when assuming different forms of parallel processing. Note that although theoretical models are explained in terms of response time (RT) pattern, the same logic also applies to error rates. **(A)** Illustration of the response-selection bottleneck (RSB) model as explanation for severe dual-task processing limitations (Pashler, 1994). Each task consists of different processing stages (i.e., P, perception; RS, response selection; MR, motor response). Processing in some stages can occur in parallel (in white). Processing of other critical stages cannot occur simultaneously (shaded), because they rely on the same capacity-limited processing channel. When both tasks overlap substantially (e.g., short stimulus onset asynchrony, SOA), Task 2 (T2) processing is interrupted, because RS2 processing has to wait until RS1 processing is completed (psychological refractory period, PRP). At long SOA, no interruption occurs, as critical stages do not overlap. This results in the typical pattern of performance decrements in T2 at short SOA (high dual-task load) compared to long SOA (low dual-task load). Task 1 (T1) processing is only little affected by temporal task overlap. **(B)** Crosstalk refers to the observation that T2 processing impacts on T1 processing, which has been taken as evidence for parallel processing despite an assumed RSB. Crosstalk effects are typically measured in response latency in T1 (RT1). The impact of T2 processing on central stage processing in T1 can be both beneficial or costly with decreasing or increasing RT1, respectively (e.g., Koch and Prinz, 2002). Importantly, any influence of T2 processing on T1, shortening or prolonging RT1, will back-propagate onto T2 (Ferreira and Pashler, 2002; Miller and Reynolds, 2003; Schubert et al., 2008). Changes in RT1 due to crosstalk should thus also be obtainable in response latency in T2 (RT2). Theoretically, crosstalk effects are not compatible with classical models of a single-channel theory (e.g., RSB model) and favor explanations in terms of capacity sharing (see **C**). However, assumptions of serial processing according to the RSB model can be preserved when assuming that different sub-components of RS2 can operate in parallel. Some authors thus distinguish response activation (RA) processes from more classical response-selection processes as the basis for interacting central components between two tasks (Hommel, 1998; Lien and Proctor, 2002; Schubert et al., 2008). **(C)** Capacity models assume that the central bottleneck is not immutable but flexible. The processing limitation arises, because two central processes require access to the same cognitive resources. Available resources are divided between the two tasks for the period during which both central stages overlap. The allocation of resources to the tasks at hand depends on task factors (e.g., instruction, incentives). Extreme forms can mimic a central bottleneck, with 100% resources allocated to T1 and 0% to T2. The more resources are shared between the two tasks (e.g., 70/30 or 50/50), the higher the RT1 increase and RT2 decrease at short SOA. This resource allocation is assumed to be realized by mechanisms of cognitive control (for details, see text).

Performing two or more tasks at the same time typically results in severe performance costs in terms of increased response latencies and/or error rates (Welford, 1952; Kahneman, 1973; Pashler, 1994). On a theoretical level, these dual-task costs have often been explained by means of a structural capacity limitation in cognitive processing. Early work on multitasking, framed within the information processing theory, assumed that access to this single processing channel is scheduled sequentially, one task at a time. For example, when a first task (T1) enters the capacity-limited processing stage, processing of an additional task (T2) is put to a halt until T1 critical stage processing is finished (see Figure 1). Following this logic, serial task scheduling is the consequence of a capacity-limited processing bottleneck that is structural in nature (Welford, 1952; Broadbent, 1958). This view of a structural limitation and a passive bottleneck-scheduling process is the core assumption of the influential and to date still widely accepted response-selection bottleneck (RSB) model (Pashler and Johnston, 1989; Pashler, 1994). Following the stage logic of cognitive processing (Sternberg, 1969), peripheral processing stages of two tasks (e.g., perception, motor response) proceed in parallel. Capacity limitation arises at central processing stages (e.g., response selection) that do not proceed at the same time (see Figure 1A, Pashler, 1984; Pashler and Johnston, 1989)^2^. This view of a structural capacity limitation for central processing stages is still prevalent in human cognitive neuroscience and textbook psychology, most likely due to the observation that even the simplest and/or highly trained cognitive operations are subject to substantial processing limitations when combined with another task (e.g., Levy et al., 2006). However, there is less consensus about whether this processing bottleneck reflects a structural (Pashler, 1998) or a strategic (Meyer and Kieras, 1997) limitation.

This theoretical assumption that central-processing limitations cause dual-task costs has largely been derived from experimental paradigms in which two choice reaction tasks are presented with varying temporal intervals (i.e., stimulus onset asynchronies, SOAs) between the stimulus of T1 (S1) and the stimulus of T2 (S2; e.g., Pashler, 1984; Pashler and Johnston, 1989; Schubert, 1999). The temporal task overlap (and thus dual-task load) can be experimentally manipulated to examine dual-task costs and thus to assess the microstructure of dual-task interference (Pashler and Johnston, 1989; McCann and Johnston, 1992; Fischer et al., 2007; see Pashler, 1994, 1998; Meyer and Kieras, 1997, for reviews).

While performance in Task 1 has been assumed to be unaffected by the manipulation of temporal task overlap, performance in Task 2 has been hypothesized to critically depend on the temporal proximity of both tasks (Figure 1A). The larger the temporal overlap between both tasks (the shorter the SOA between S1 and S2), the slower the responses and higher the error rates in Task 2. The difference in response time in Task 2 (RT2) between short and long SOA has been called the *psychological refractory period* (PRP) effect (Pashler, 1994; Meyer and Kieras, 1997). The PRP effect reflects a widely used measure of dual-task costs evolving at the capacity-limited response-selection stage in dual-tasking (Figure 1A). Importantly, these dual-task costs (i.e., performance increments at high temporal task overlap compared to low temporal task overlap) serve as marker for multitasking efficiency.

### Demonstrating Parallel Processing Under the Assumption of Limited Resources

Within the PRP paradigm, the assumption of parallel processing means that central cognitive processing in T2 can proceed in parallel to central capacity-limited stage processing in T1 (Figures 1B,C). Structural bottleneck models deny the possibility of central T2 processing during the bottleneck (i.e., the PRP). Yet, parallel T2 processing during the T1 bottleneck stage has been demonstrated with two different approaches, yielding to two distinct result patterns, i.e., the *locus of slack logic* and the *backward crosstalk logic*.

In the locus of slack logic (Pashler and Johnston, 1989; McCann and Johnston, 1992), the possibility of parallel processing is assessed in performance measures of T2. For this, the duration (e.g., difficulty) of a central cognitive process in T2 is manipulated. The relationship between easy and difficult conditions and SOA is particularly important in these cases. If no difficulty differences are obtained at short SOA, an absorption into the bottleneck (and hence parallel processing during the bottleneck) is assumed. If the manipulated central process cannot proceed during the bottleneck, the duration/difficulty manipulation should reveal additivity with the SOA manipulation. The manipulation should be visible to the same extent at short and long SOA (for successful demonstrations of parallel central processing using locus of slack, see Oriet et al., 2005; Fischer et al., 2007; Fischer and Schubert, 2008; Janczyk, 2013).

Backward crosstalk logic is based on the empirical finding that processing of two tasks rarely occurs independently (Navon and Miller, 1987). Especially in conditions of high temporal proximity (i.e., short SOA) and high task similarity (i.e., both tasks share dimensional overlap), the likelihood of between-task interaction is increased. Figure 1B illustrates the possibility of this *crosstalk*. Central T2 task processing affects central T1 processing prior to completion of Task 1 bottleneck-stage processing (Duncan, 1979; Navon and Miller, 1987, 2002; Hommel, 1998; Logan and Schulkind, 2000; Koch and Prinz, 2002; Miller, 2006; Fischer et al., 2007). As a consequence, any T1 processes subjected to the central bottleneck are either facilitated or prolonged. In contrast to the locus of slack logic, evidence for parallel processing based on crosstalk logic therefore comes from modulations of performance measures in T1 caused by central T2 processing.

A seminal study using this logic was provided by Hommel (1998). In his experiment, participants responded to the color (red/green) of letters in T1 and to their identity (H/S) in T2. Crosstalk effects on RT1 were demonstrated when response codes for both tasks overlapped. More specifically, manual T1 responses to letter colors (e.g., red-*left* and green-*right* response) and verbal T2 responses to letter identity (e.g., saying *left* to an H and *right* to an S) produced substantial crosstalk interference (RT1 prolongation), when the verbal answer *right* coincided with the *left* manual response (response-category mismatch). T2 processing facilitated T1 response activation (RT1 decrease), when the verbal answer *right* was accompanied with the *right* manual response (response-category match). S-R translation processes of T1 and T2 were not serial and discrete but proceeded in parallel, when dimensional overlap between tasks was provided (see also Logan and Schulkind, 2000; Koch and Prinz, 2002; Miller and Alderton, 2006; Fischer et al., 2007; Schubert et al., 2008; Janczyk et al., 2014). This finding of significant central T2 processing during the PRP challenges single-channel theories of serial processing (Pashler, 1984). Instead, the observed result pattern is in line with a capacity sharing assumption. The finding of crosstalk can however be reconciled with the notion of the RSB model, when adding a distinction between response activation (RA) and response identification (RI). While the former features no capacity limitations, thus allowing for crosstalk, the latter requires all central resources, causing a bottleneck (Hommel, 1998; Lien and Proctor, 2002; Schubert et al., 2008).

Further evidence for parallel central processing in dual tasks comes from electrophysiological studies. Behavioral crosstalk effects, as reported in Hommel (1998), are accompanied by deflections in the lateralized readiness potential (LRP) in T2, reflecting T2 RA processes that start prior to completion of central bottleneck processing in T1 (Lien et al., 2007). Similarly, measuring the LRP during T1 processing, Ko and Miller (2014) showed that the time between S1 onset and onset of the stimulus-locked LRP was modulated by central T2 processing, thus providing further evidence for parallel central T2 processing.

### Parallel Processing Without the Assumption of Limited Resources

The strict notion of limited resources and the consequential serial processing has been questioned on both experimental and theoretical grounds. It can be traced back to early attentional-filter conceptions of Deutsch and Deutsch (1963) and the assumption of multiple specialized resources (Norman and Bobrow, 1975; Wickens, 1984; but see Allport, 1980).

Early work in the 1970s identified factors such as task similarity and task practice that crucially determine the possibility of parallel task processing. For example, Allport et al. (1972) argued that dual-task costs do not result from exceeding the capacity of a single-channel processor but from the difficulty of separating two similar tasks. When combining two highly dissimilar tasks (e.g., repeating continuous speech and sight-reading difficult piano music), piano players were able to demonstrate parallel processing of both tasks with a quality comparable to single-task processing (Allport et al., 1972; see also Shaffer, 1975; Wickens, 1984). Similarly, Shaffer (1975) showed that participants skilled in typewriting can easily perform copy-typing (typing from a sheet) with a verbal shadowing task in parallel, but fail in combining an audio-typing task with reading from a sheet (for more recent research on the role of modality pairings when determining dual-task costs, see Stelzel et al., 2005; Hazeltine and Ruthruff, 2006; Hazeltine et al., 2006; Huestegge and Koch, 2013; Halvorson and Hazeltine, 2015). Although the aforementioned early studies revealed impressive capabilities in multitasking, many of these studies have been criticized, because their task timing allowed for fast switches between task-component processing (e.g., multiplexing). It is thus conceivable that the results only mimic parallel processing instead of actually representing it.

Another demonstration of parallel processing was achieved by administering a large amount of dual-task practice. Spelke et al. (1976) trained two participants to read short stories, while writing lists of words at dictation. Training took part over a period of 17 weeks (with five 1-h sessions per week). After the training, dual-task performance approached the quality of individual single-task performance. Because people easily develop skills through practice, the authors postulated that the potential of skill acquisition in any domain might question the general concept of limited cognitive capacity. In 2001, the assumption of parallel processing by extensive practice received a revival, mainly due to the implementation of timing- and interference-controlled dual-task paradigms. In an influential study, Schumacher et al. (2001) presented two choice reaction tasks (e.g., a visual-manual and an auditory-vocal task) simultaneously (SOA = 0 ms) and with equal task priority. Multitasking efficiency was captured in the extent to which performing each task in a dual-task context equals single-task performance. The criterion of perfect time sharing (dual-task performance equals single-task performance) was achieved after only five practice sessions (see also Ruthruff et al., 2001; Hazeltine et al., 2002; Oberauer and Kliegl, 2004; Liepelt et al., 2011a,b; Strobach et al., 2012). Proponents of the RSB model however argued that practice leads to shortening of processing stages which, in turn, reduces the effects of the bottleneck instead of bypassing it (e.g., latent bottleneck assumptions, see, e.g., Ruthruff et al., 2003; Dux et al., 2009).

### Theoretical Models Allowing for Parallel Processing

Resource models represent a major group of theoretical models that allow for the possibility of parallel task processing. In line with single-channel theories, they also assume a strict capacity limitation in central cognitive processing. In contrast to the RSB model however, they incorporate the idea that the available somewhat limited resources can be scheduled and allocated to specific task processing (Kahneman, 1973; Norman and Bobrow, 1975). An extension of the assumption of an *unspecific central* resource (Kahneman, 1973) is the idea of *multiple specific* resources (Wickens, 1984, 2002). Under the assumption of multiple resources, parallel processing can occur, for example, if task components are scheduled in different processing threads that are coordinated and scheduled by different resources (e.g., Salvucci and Taatgen, 2008).

According to resource models, dual-task costs arise, because processing of different task components requires the same limited resources (Kahneman, 1973; Navon and Miller, 2002; Tombu and Jolicoeur, 2003). In these cases, resources are allocated in an all or none fashion. The classical PRP effect would reflect an extreme form of resource sharing of 100% of resources to T1 and 0% to T2, mimicking a capacity-limited central processing bottleneck due to the instruction of T1 priority. In contrast to RSB models, limited processing resources can however be shared between two tasks in varying proportions (e.g., 80% T1 and 20% T2). The more resources are shared, the more parallel processing occurs. With this logic, capacity-sharing models incorporate the central bottleneck assumption (Navon and Miller, 2002; Tombu and Jolicoeur, 2002, 2003; Lehle and Hübner, 2009) and account for often observed findings of between-task crosstalk in PRP-like paradigms (Hommel, 1998; Logan and Schulkind, 2000; Koch and Prinz, 2002; Miller, 2006; Fischer et al., 2007; Schubert et al., 2008; Koch, 2009).

The proposition of a flexible allocation of resources requires a definition of how attentional resources are distributed. Arguments have been made that the allocation of attentional resources is not incidental but depends on instructions (Lehle and Hübner, 2009; Lehle et al., 2009), task priority, and outcome value (Wickens et al., 2003). It reflects strategic resource scheduling, typically realized by cognitive control processes (Meyer and Kieras, 1997; Logan and Gordon, 2001; Sigman and Dehaene, 2006). The assumption of strategic and flexible allocation of processing capacity is supported by recent functional magnetic resonance imaging (fMRI) findings indicating that brain areas associated with cognitive control are activated during dual-tasking (e.g., Szameitat et al., 2002; Marois and Ivanoff, 2005; Dux et al., 2006; Stelzel et al., 2009; Tombu et al., 2011). In addition, a recent fMRI study adopting a dual-task paradigm introduced by Miller et al. (2009) to create conditions of more serial versus more parallel task processing (for details, see below) provided evidence for distinct neuroanatomical correlates of response selection depending on task-processing constraints. In more detail, under conditions of increased parallel task processing response selection mechanisms operated at the striatal level, whereas under conditions of increased serial task processing response selection was accompanied by primarily lateral prefrontal cortex activation (Yildiz and Beste, 2014).

Theories that postulate the involvement of cognitive control functions to explain limitations in multitasking do not necessarily feature assumptions of general limited resources. Multitasking limitations occur due to competing processes that require access to the same local and task-specific resources (Navon and Gopher, 1979). This notion is important, because instead of indicating structural constraints of limited capacity, the observed limitations reflect functional constraints (e.g., computational limitation). Functional constraints arise, when the same representation is used “for different purposes by multiple processes” (Feng et al., 2014, p. 130). For example, in cognitive control theories of dual tasking such as the executive control of visual attention (ECTVA) theory (Logan and Gordon, 2001), the observation of serial task processing results from the effort to avoid interactions between multiple task-component processing (e.g., between stimulus-response bindings). This has been formulated in terms of the *dual-task binding problem* that describes the challenge to correctly map stimuli of each task (S1 and S2) onto the correct responses in each task (R1 and R2 for Task 1 and Task 2, respectively). To solve the binding problem and therefore to minimize the risk of response reversals and/or confusion, a strategy of serial processing is adopted (Logan and Gordon, 2001). The cognitive system can flexibly respond to requirements of the dual-task situation (Meyer and Kieras, 1997) by adopting a sequential processing strategy, if the risk of task reversals is high. As a consequence, serial processing of two tasks appears to be the more efficient processing strategy in general (Logan and Gordon, 2001; Miller et al., 2009) with the functionality of reducing between-task interference (Navon and Miller, 1987; Logan and Gordon, 2001; Tombu and Jolicoeur, 2003; Lehle and Hübner, 2009; Oberauer and Bialkova, 2011). Choosing a serial processing mode thus reflects a functional and strategic *option* (see also Hazeltine et al., 2008).

## Why Do Individuals Employ a Parallel Task-Processing Mode in the Face of Increased Between-Task Interference?

We have established that individuals are able to adopt a strategy of parallel task-component processing when performing more than one task at a time. Given that parallel processing is associated with increased risks of between-task interference and is less efficient, why should participants opt for this processing strategy in the first place? Miller et al. (2009) provided a first answer to this question by showing that parallel processing has the means to outperform serial processing in terms of dual-task efficiency. They defined dual-task efficiency as the total time that it takes to complete two tasks (RT1 + RT2 = total reaction time, TRT). In two experiments, the PRP logic with two independent tasks was applied and the ratio of short to long SOA was varied in a list-wide proportion-SOA manipulation, i.e., participants performed lists with mostly short SOAs and lists with mostly long SOAs. The authors not only showed that lists with mostly short SOAs produced typical result patterns of parallel processing (i.e., increased RT1 and decreased RT2, Figure 1C) but also revealed that list-wide SOA manipulations determined the efficiency of parallel and serial processing modes. Importantly, in lists with mostly short SOAs parallel processing turned out to be more efficient than serial processing. In typical PRP paradigms the temporal proximity between tasks (SOAs) is rather balanced. Thus, a serial processing strategy would seem favorable there.

Another reason for the fact that we mostly observe serial processing when testing participants with typical PRP-like dual-task paradigms in our laboratories is the strong T1 priority instruction. Strong emphasis on T1 performance favors a resource allocation that primarily or even entirely benefits T1 processing (e.g., 100% of available resources are allocated onto T1). Accordingly, result patterns that speak for serial processing are not surprising. Yet, when specific priority instructions are lifted, participants freely choose a moderate parallel processing strategy (Lehle and Hübner, 2009), resulting in increased crosstalk between tasks (Figure 1B). An explanation of why participants may adopt a less efficient processing mode was put forward by Lehle et al. (2009), showing that parallel processing is associated with less mental effort, as confirmed by reduced levels of peripheral physiological measures and subjective effort ratings in parallel compared to more serial processing. Therefore, although parallel processing is not the most efficient way of dual-task processing (in most cases), it seems to reflect a less effortful processing strategy compared to strict serial processing. Given the choice, participants seem to adopt the processing mode of least mental effort (Hull, 1943; Kool et al., 2010).

## Shifting Between Parallel and Serial Processing Modes as Marker of Adaptive Behavior

Even though the question of whether and to what extent parallel task processing is possible in dual-task performance is frequently discussed in the literature (Han and Marois, 2013), it might not be what we cognitive psychologists need to ask ourselves in this context. Instead, in our opinion, two key questions to guide current discussions and future research efforts result from the work as summarized above: *(1) How is the shift between these complementary dual-task processing modes realized? (2) Under which conditions are parallel and serial processing the more adaptive and thus favorable choice?* As a reply to the first question, we propose that optimized and efficient multitasking reflects the ability to flexibly adopt an either parallel or serial task-processing mode, depending on situational demands. Multitasking requires an individual to maintain a balance between two antagonistic types of performance optimization, i.e., minimizing between-task interference (by increasing *serial* task processing) and minimizing mental effort (by allowing for more *parallel* processing). Flexibly adjusting the degree of more serial versus more parallel dual-task processing to changing task and context requirements reflects high levels of adaptability in dynamic environments including the online analysis of contextual features, their translation into performance requirements, implementation of the optimal processing mode, and continuous monitoring of both the environmental demands and performance outcome as basis for further tuning, where required (Goschke, 2013; Fischer et al., 2014).

Raising the question as to whether participants can top-down control their multitasking processing mode, recent studies provide evidence that individuals are able to implement substantial block-by-block switches between more parallel and more serial task-processing modes while continuing to perform the same task when instructed to do so (Lehle and Hübner, 2009). In the framework of ECTVA (Logan and Gordon, 2001), instructions are assumed to define a set of cognitive control parameters (e.g., task priority, attentional breath, etc.) which, applied to a dual-task context, means that they determine the degree of serial versus parallel processing. On the cognitive process level, the extent of serial task processing might be then translated into two aspects, i.e., (a) the prioritization of Task 1 processing (Stelzel et al., 2009) and (b) a temporary inhibition of additional Task 2 processing (Koch et al., 2010).

In addition to the top-down regulation, there is evidence for context-driven shifts between more parallel versus more serial dual-task processing. For example, the aforementioned expectation of the temporal overlap between two tasks (Miller et al., 2009) and task difficulty determine the degree of parallel versus serial task processing (e.g., task difficulty induces a bias toward increased serial dual-task processing which is reflected in steeper RT2 declines with increasing SOA; Luria and Meiran, 2005; Fischer et al., 2007). Recently, we also provided evidence for a complete bottom-up adjustment of parallel versus serial processing (Fischer et al., 2014). Using a crosstalk approach (Figure 1B), we tested whether participants are able to extract statistical contingencies (i.e., the probability of between-task interference) from the task context and use this information to adjust the amount of parallel versus serial task processing. More specifically, dual-task conditions with high risks of crosstalk interference (80% crosstalk interference trials) and dual-task conditions with low risks of crosstalk interference (20% crosstalk interference trials) were presented at distinguished locations on the screen. Although overall likelihood of crosstalk interference was 50/50, participants were able to adjust the strength of task shielding (by means of increased serial processing) in a location-specific manner. The extent of serial processing was significantly increased for the location with high likelihood of crosstalk interference, which resulted in reduced crosstalk for trials at that location. In trials presented at the location with low interference likelihood, shielding was reduced and higher levels of parallel processing were observed. Therefore, if a context requires more protection of T1 processing from T2 influences, task shielding is increased, resulting in less parallel processing.

Studying impacting factors of additional task performance on prioritized motor movements, we demonstrated that the preceding trial history (i.e., conflict between tasks in the previous trial) influences the balance between parallel and serial task processing (Scherbaum et al., 2015). Using continuous motor-execution tasks (i.e., mouse movements in both tasks), we showed that large amounts of crosstalk interference during the previous trial (N-1) resulted in the adoption of a more serial task-processing mode, as evidenced by reduced crosstalk interference in Trial N. Such sequential modulations are typically accounted for by response conflicts triggering the recruitment of cognitive control (Gratton et al., 1992; Botvinick et al., 2001). In the dual-task context, this reflects a bias toward increased serial task processing to reduce crosstalk interference.

We further demonstrated that the balance between complementary dual-task processing modes is determined by internal situational features such as variations in an individual’s mood, task-preceding cognitive control state, or acute stress levels. First, we administered a mood-induction procedure (with controlled arousal effects) prior to a dual task with crosstalk. Participants that underwent a negative mood-induction protocol displayed higher levels of serial task processing (e.g., less between-task interference) than participants that underwent positive mood induction (Zwosta et al., 2013). Second, we manipulated the level of parallel versus serial processing by activating different cognitive control states prior to dual-task performance (Fischer and Hommel, 2012). Participants solved different types of creativity tasks associated with either cognitive flexibility (i.e., divergent thinking) or cognitive persistence (i.e., convergent thinking) prior to the dual-task session. In the convergent-thinking group, participants performed an adapted version of the remote association test (RAT; Mednick, 1962), in the divergent-thinking group, participants performed a version of the alternative uses task (AUT; Guilford, 1967) in order to induce convergent versus divergent thinking, respectively. The convergent-thinking group displayed stronger serial processing to reduce between-task interference compared to both divergent-thinking and control group. Third, we investigated the effect of acute stress on dual-task processing modes. Following an acute psychosocial stressor (Trier Social Stress Test), participants adopted a more resource-saving processing strategy of increased parallel processing compared to controls without the stress experience (Plessow et al., 2012). Stressed participants allowed for increased levels of between-task interference, presumably because a strict sequential and serial scheduling of task processing would be too effortful and resource-demanding. We interpreted our finding as a sacrificing and compensatory strategy in order to maintain overall high dual-task performance. The notion that parallel processing reflects a more resource-saving processing mode and is mentally less demanding (Lehle et al., 2009) fits nicely with our observation of compensatory cognitive strategy changes as a result of acute stress experience. Using a different dual-task paradigm consisting of two independent tasks without the possibility of between-task interactions, stress-related compensatory strategy shifts entailed an increased T1 priority focus, speeding T1 component processing and, as a result, reducing critical bottleneck stage processing (Beste et al., 2013).

These examples illustrate that the adoption of a more parallel or a more serial task-processing mode largely depends on the conditions under which multiple tasks are performed. With this, the situation-dependent implementation of complementary task-processing modes in multitasking can be integrated into a general framework posting the regulation of cognitive control as fundamental basis underlying adaptive goal-directed behavior (Cohen et al., 2004, 2007; Goschke, 2013). Adaptive action control requires the dynamic adjustment between complementary control demands. While task-irrelevant stimuli need to be ignored and blocked from being processed in order to prevent interference with task-relevant processing (goal shielding), complete shielding is dysfunctional and even potentially harmful, as it would prevent the individual from monitoring the environment for potentially relevant stimuli (background monitoring) that may signal a change in action goal (e.g., stimuli that imply danger). Instead, a dynamic regulation of these complementary control processes is required, reflecting a tradeoff between antagonistic constraints (see Goschke, 2003, 2013; Goschke and Bolte, 2014, for an overview). Such “control dilemmas” describe the need to be continuously and flexibly adjusted based on (changing) environmental demands (Goschke, 2003; Cohen et al., 2004, 2007). A recent argument is that dysfunctional control parameter settings in terms of sustained biases toward one particular (often extreme) control state might represent an endophenotype of a variety of mental disorders (e.g., Goschke, 2014).

Such extreme biases and their behavioral consequences have been primarily investigated in healthy populations. For example, an experimentally induced bias toward high stability comes at the cost of reduced cognitive flexibility (Dreisbach and Goschke, 2004; Plessow et al., 2011; Fischer and Hommel, 2012), which, if maintained, might not only turn out to be dysfunctional in situations calling for cognitive flexibility but additionally manifest in overall cognitive rigidity and compulsive behavior (Meiran et al., 2011). A key future scientific endeavor will be to determine the cognitive mechanisms that underlie the flexible adoption of complementary task-processing modes in general.

Applying this framework, multitasking in itself constitutes a prime control dilemma, in which a crosstalk-avoiding serial processing strategy counters an effort-saving parallel processing strategy. Complete T1 shielding and thus T2 blocking is dysfunctional, as successful multitasking requires the attendance and processing of T2 components. Yet, the efficiency of adaptive multitasking might be seen in the flexible selection of a situation-adequate processing strategy within a *continuum* from serial to parallel task processing. Such a conception also has direct consequences for defining efficient multitasking, and it raises the question of how to optimize the flexible and context-sensitive adoption of complementary task-processing modes.

We would like to conclude this discussion about parallel and serial processing in multitasking by emphasizing that the inclusion of closely related topics into further investigations of multitasking will be crucial as additional source to inform our understanding of efficient multitasking and its underlying cognitive processes. First of all, incorporating an *individual difference* perspective on multitasking ability in general and the adaptive adjustment of task-processing strategies in particular might hold promise for gaining novel insights into factors of optimized multitasking performance. For example, individuals frequently engaging in multitasking are not necessarily the ones displaying efficient multitasking performance (Ophir et al., 2009; Sanbonmatsu et al., 2013). In fact, multitasking activity correlated negatively with multitasking ability but positively with impulsiveness and sensation seeking (Sanbonmatsu et al., 2013). Individuals that frequently multitask (e.g., heavy media multitaskers) are more easily distracted by irrelevant information and less able to focus on a single goal (Ophir et al., 2009). In the context of the outlined discussion, this behavior can be framed as a task-processing bias continuously tuned toward heightened levels of parallel processing. Another promising finding in individual differences in multitasking abilities that warrants further investigation is the detection of “supertaskers,” i.e., individuals who do not show performance decrements in multitasking compared to single-task performance (Watson and Strayer, 2010). Their ability has been explained in terms of a more efficient recruitment of cognitive control and an increased ability to maintain and coordinate multiple goals and limitations in information processing, enabling supertaskers to more effectively deal with situations of heightened cognitive load (Medeiros-Ward et al., 2014).

Secondly, research contrasting tentative processes underlying multitasking with analogous processes in related fields of the study of attentional limitations, e.g., to more clearly determine at which points more serial and more parallel task processing are more adaptive and efficient (see, for example Miller et al., 2009). To illustrate this point, unified bottleneck theories propose close similarities between attentional limitations in sensory consolidation as in the attentional blink (Raymond et al., 1992) and attentional limitations in response selection as in the PRP paradigm (Tombu et al., 2011; Marti et al., 2012; Garner et al., 2014). Yet, while PRP research often aims at optimizing dual-task performance by increasing the engagement of cognitive control (e.g., through an emphasis on speed plus reward), it has been shown that limitations in the attentional blink arise from an overinvestment of cognitive control (Olivers and Nieuwenhuis, 2006; Taatgen et al., 2009). Therefore, future research is needed to determine whether strategies of increasing versus decreasing the engagement of cognitive control might be promising when aiming at reducing dual-task costs in standard training protocols.

Thirdly, an important question to guide future research in this area is in which respect the ability to flexibly adopt complementary task-processing modes is related to fluid intelligence, further specifying the currently investigated link between core executive control competencies and fluid intelligence as well as flexible adaptation to environmental changes as a hallmark of fluid intelligence (Duncan et al., 2008; Jaeggi et al., 2008; Duncan, 2010; Diamond, 2013; Au et al., 2015).

## Conclusion

Starting from the question of what constitutes adaptive multitasking performance, we reviewed empirical evidence for two processing modes in multitasking that are not mutually exclusive, i.e., serial versus parallel task-component processing. Demonstrating that parallel task processing is indeed possible when performing more than one task at a time has challenged the view that the frequently observed multitasking costs represent an inevitable consequence of a structural capacity limitation. Instead, it suggests that these multitasking costs may signal a functional limitation (e.g., with the purpose of avoiding crosstalk). In the second part of the review, we highlighted that evidence for parallel processing critically depends on the theoretical and methodological basis under which multitasking performance is assessed.

While serial task processing appears to be the most efficient multitasking processing strategy, participants are able to adopt parallel processing. Moreover, parallel processing can even outperform serial processing under certain conditions. Based on these highlighted insights into multitasking performance, future research aiming to further understand the nature of parallel versus serial processing of multiple tasks to unveil the secrets of multitasking efficiency needs to take into account the preconditions and environmental constraints under which multitasking is performed. We believe that a flexible and context-sensitive recruitment of a more serial or more parallel processing strategy enables the agent to flexibly adjust to environmental demands, providing important mechanisms for adaptive intelligent behavior (Cohen et al., 2004, 2007; Goschke, 2013).

### Conflict of Interest Statement

The authors declare that the research was conducted in the absence of any commercial or financial relationships that could be construed as a potential conflict of interest.

## Footnotes

1 In more detail, serial processing means strict sequential processing of certain critical (e.g., capacity-demanding) cognitive processes due to the assumed bottleneck. When a cognitive process A is underway, another cognitive process B cannot operate at the same time. For the assumption of parallel processing, however, processes A and B proceed simultaneously (at least to certain degrees), i.e., in parallel to each other and despite the capacity limitation of the bottleneck.

2 It should be noted that there are alternative conceptions of the bottleneck theory, that mainly differ in the location of the capacity-limited processing stages, proposing either motor execution bottlenecks (e.g., Keele, 1973; Ulrich et al., 2006; Bratzke et al., 2009) or multiple bottlenecks in the information-processing stream (De Jong, 1993).
